# Development of a core set of gait features and their potential underlying impairments to assist gait data interpretation in children with cerebral palsy

**DOI:** 10.3389/fnhum.2022.907565

**Published:** 2022-10-20

**Authors:** Marjolein M. van der Krogt, Han Houdijk, Koen Wishaupt, Kim van Hutten, Sarah Dekker, Annemieke I. Buizer

**Affiliations:** ^1^Department of Rehabilitation Medicine, Amsterdam UMC, Vrije Universiteit Amsterdam, Amsterdam, Netherlands; ^2^Amsterdam Movement Sciences, Rehabilitation & Development, Amsterdam, Netherlands; ^3^Department of Human Movement Sciences, Faculty of Behavioral and Movement Sciences, Vrije Universiteit Amsterdam, Amsterdam, Netherlands; ^4^Department of Human Movement Sciences, University Medical Center Groningen, University of Groningen, Groningen, Netherlands; ^5^Heliomare Rehabilitation Centre, Wijk aan Zee, Netherlands; ^6^Basalt Rehabilitation Centre, The Hague, Netherlands; ^7^Emma Children’s Hospital, Amsterdam University Medical Center, Vrije Universiteit Amsterdam, Amsterdam, Netherlands

**Keywords:** clinical gait analysis, gait interpretation, impairment focused interpretation, clinical reasoning, interpretation tool, cerebral palsy, rehabilitation, biomechanics

## Abstract

**Background:**

The interpretation of clinical gait data in children with cerebral palsy (CP) is time-consuming, requires extensive expertise and often lacks transparency. Here we aimed to develop a set of look-up tables to support this process, linking typical gait features as present in CP to their potential underlying impairments.

**Methods:**

We developed an initial core set of gait features and their potential underlying impairments based on biomechanical reasoning, literature and clinical experience. This core set was further specified through a Delphi process in a multidisciplinary group of experts in gait analysis of children with CP and evaluated on 20 patient cases. The likelihood of the listed gait feature–impairment relationships was scored by the expert panel on a five-point scale.

**Results:**

The final core set included 120 relevant gait feature–impairment relations including likelihood scores. This set was presented in the form of look-up tables in both directions, i.e., sorted by gait features with potential underlying impairment, and sorted by impairments with potential related gait features. The average likelihood score for the relations was 3.5 ± 0.6 (range 2.1–4.6).

**Conclusion:**

The developed set of look-up tables linking gait features and impairments, can assist gait analysts and clinicians in standardized biomechanical reasoning, to support treatment decision-making for gait impairments in children with CP.

## Introduction

Gait analysis is often performed in children with cerebral palsy (CP) to diagnose gait problems, assist in treatment decision making, and to evaluate treatment outcomes, with the goal to improve walking function in daily life. Walking problems occur frequently in children with CP, and gait can be affected in various ways by a multitude of underlying neural and mechanical impairments such as spasticity, muscle weakness, joint or muscle contractures, or bony deformities.

The interpretation of gait analysis data is complex, as it comprises a large amount of information, including qualitative information from videos, quantitative data on kinematics, kinetics and electromyography (EMG), and complimentary data from physical exam and imaging. Gait analysis is a multifaceted and multidisciplinary process, in which all available elements are taken into account and interrelated to understand the origin of the gait problems and decide on the targets for treatment. In literature, there is much focus on gait data acquisition methods such as marker models (Leardini et al., 2017) and reproducibility (McGinley et al., 2009), but the process of interpretation of gait data has deserved far less attention. It has been shown that interpretation can differ substantially between centers, leading to inconsistent treatment recommendations for the same subject (Skaggs et al., 2000; Wright, 2003). Therefore, using standardized methods for data interpretation is important to ensure complete, consistent, transparent and reproducible conclusions from gait analysis (Simon, 2004).

“Impairment focused interpretation” is one standardized way of gait data interpretation (Baker, 2013). This method focuses on identifying neuromechanical impairments underlying gait deviations, which, combined with environmental and personal factors as well as other aspects that may affect walking, are used by the clinician to come to a treatment decision. The suggested process starts with making a complete list of deviating kinematic gait features in a systematic manner, typically done for each leg separately. The next step is to relate these features to each other and to underlying neural or mechanical impairments that explain the gait deviations present. Although this method standardizes the process of neuromechanical reasoning, the second step of combining gait features and linking these to underlying impairments is challenging. Gait features can be related to multiple impairments and the selection of relevant underlying impairments by the assessor is often not explicit, making interpretation subjective and not transparent.

Nevertheless, it is evident that with experience, it becomes easier to identity patterns of gait features that can be caused by a certain impairment, and to identify different potential causes for certain gait deviations. It would be worthwhile to make this expert knowledge more explicit and, combined with literature data, explicitly formulate the potential relationships between gait features and potential impairments. This would not only help novices in acquiring the skill of gait data interpretation, but also allow for better standardization, more transparency, and validation of this process. Therefore, the aim of this study was to develop an explicit core set of gait features and their potential underlying impairments as present in CP, based on literature, biomechanical reasoning and clinical expertise, to support an impairment-focused interpretation approach.

## Methods

**FIGURE 1:**
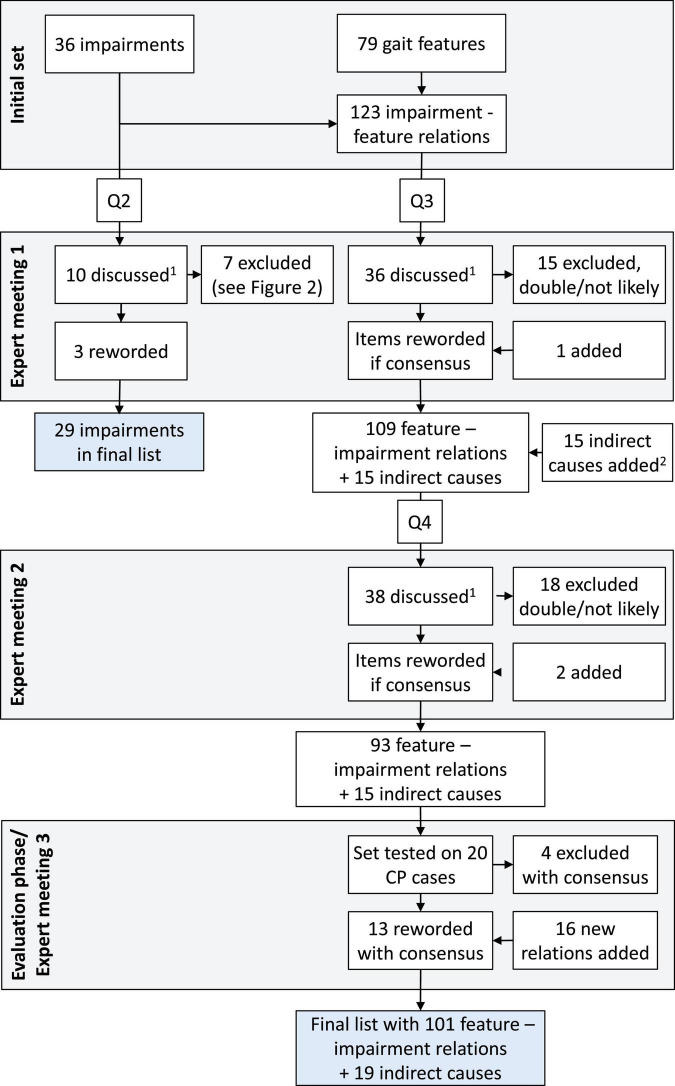
Flow chart of the process of questionnaires (Q2-4) and expert meetings. Q1 is not included, as this questionnaire only asked for background and expertise of the panelists. ^1^All items with low likelihood scores (< 2, likely not relevant), a large difference between panelists (range > 3, possibly unclear), or that received many comments were discussed. ^2^In the list of gait deviations with potential underlying causes, several “indirect causes” were also added, such as secondary effects or compensations (see Supplementary Appendix A).

For this study we chose a modified Delphi approach (Boulkedid et al., 2011), to systematically seek consensus on potential relations between gait features and their possible underlying impairments. The whole process is shown in Figure 1.

First, an initial set of gait features and potential underlying impairments was created, based on a combination of literature, own clinical expertise (i.e., a preliminary set drafted for clinical use within Amsterdam UMC) and biomechanical reasoning. Literature was searched for publications that included lists of gait feature–impairment relations, which yielded two papers that contained a substantial number of relations (Armand et al., 2016, Zhou et al., 2017). Furthermore, unpublished course material was used that included a number of possible relations. The gait features included were restricted to kinematic data typically collected in gait laboratories, including 3D motion capture and video gait data of trunk, pelvis, hip, knee and ankle joint and basic foot motion, excluding for instance head and arm motions and more detailed (internal) foot motions. Possible underlying impairments were restricted to neural and musculoskeletal impairments as often present in CP (i.e., spasticity, contracture, weakness, limited selective motor control, and bony deformities), excluding aspects such as pain, sensory deficits and motivational or psychological factors. Spasticity and contracture of the same muscle were combined into one item, as their effect on gait is difficult to distinguish based on kinematics alone. Feature–impairment relations were mostly defined within the ipsilateral leg, as gait analysis interpretation is typically done for each leg individually. However, clear causes of gait features related to the contralateral leg were included as well. Gait phases were defined following Harlaar (2014), as these include systematic phases and events (instances) in the gait cycle and are most commonly used throughout the Netherlands (see Supplementary Appendix A).

Next, a Dutch expert panel was formed for the Delphi process, in which the initial set of gait feature–impairment relations was evaluated and further specified. A total of 24 (pediatric) physiatrists, human movement scientists, physical therapists and gait analysts, with extensive knowledge and experience in pediatric clinical gait analysis, were asked to participate. They came from 10 different centers all experienced in clinical gait analysis. Panelists were selected for being renowned in the field, and/or member of the Dutch/Belgian Society for Movement Analysis Laboratories in the Low Lands (SMALLL) and/or the Netherlands Society of Rehabilitation Medicine (NSRM). For practical reasons, and to ensure all participants were familiar with the impairment focused interpretation approach, all experts were recruited from Dutch clinical centers.

All experts that were willing to participate filled in five digital questionnaires (Q1-5) and participated in three expert meetings (see Figure 1). In Q1, experts were asked about their background and experience in clinical gait analysis. In Q2, experts indicated on a 5-point scale how often they thought each impairment from the preliminary set would be present in children with CP (1 = almost never, 5 = very often). In Q3, experts indicated, using the same scale, how often each impairment would coincide with each possible related gait feature from the preliminary gait feature–impairment list. Furthermore, experts could comment in free-text comment fields on the wording or other aspects of each impairment, gait feature and their relation. In expert meeting 1, the results of Q1-3 were discussed, and items were either removed, reworded, or added, based on the consensus (> 70%) of the group.

A few weeks later, experts filled in Q4, in which they were again asked to score the likelihood of the updated gait feature–impairment relations, but now with the order reversed, i.e., grouped by feature. In expert meeting 2, the results of Q4 were discussed, and items removed, reworded or added based on consensus. In case large changes were made in meeting 2, the panelists were asked to score the likelihood of these updated relations.

The resulting set of gait feature–impairment relations was then evaluated for clarity and completeness, using a retrospective data set of 20 children with spastic CP. The children were aged 8.9 ± 3.2 years (range 5–14) and classified as gross motor function classification system (GMFCS) level I (*N* = 5), II (*N* = 12), or III (*N* = 3). For each child, the most affected leg was analyzed. 2D video recordings and 3D gait report were available for all children, as well as EMG and physical examination data. Children had not undergone an orthopedic or neurosurgical procedure in the year before the gait analysis. Written permission for the use of their data for scientific research was given. All cases were interpreted using the developed set of gait features and underlying impairments by an expert pediatric physiatrist and two students educated in clinical movement analysis.

All missing or unclear relations as gathered in this evaluation were discussed with the expert panel in expert meeting 3, held online. Finally, the resulting additional relations were presented to the panel in online questionnaire Q5 to be scored for their likelihood. This procedure resulted in a final set of gait feature–impairment relations, including their likelihood scores based on expert opinions. For each gait feature–impairment relation, the mean, standard deviation, median and range of the likelihood scores were calculated and presented.

## Results

Seventeen experts filled in all questionnaires. The final panel consisted of seven physicians, five researchers, and five therapists/gait analysts. Their average age was 48.8 ± 9.0 years (range 32–60), and their experience in clinical gait analysis was 12.4 ± 4.7 years (range 4–18). Nine panelists had experience in performing gait analysis measurements (12.1 ± 4.6 years), while all panelists had experience in gait analysis interpretation (11.8 ± 4.8 years). Fourteen panelists participated in expert meeting 1, 13 in expert meeting 2, and 16 in expert meeting 3.

**FIGURE 2:**
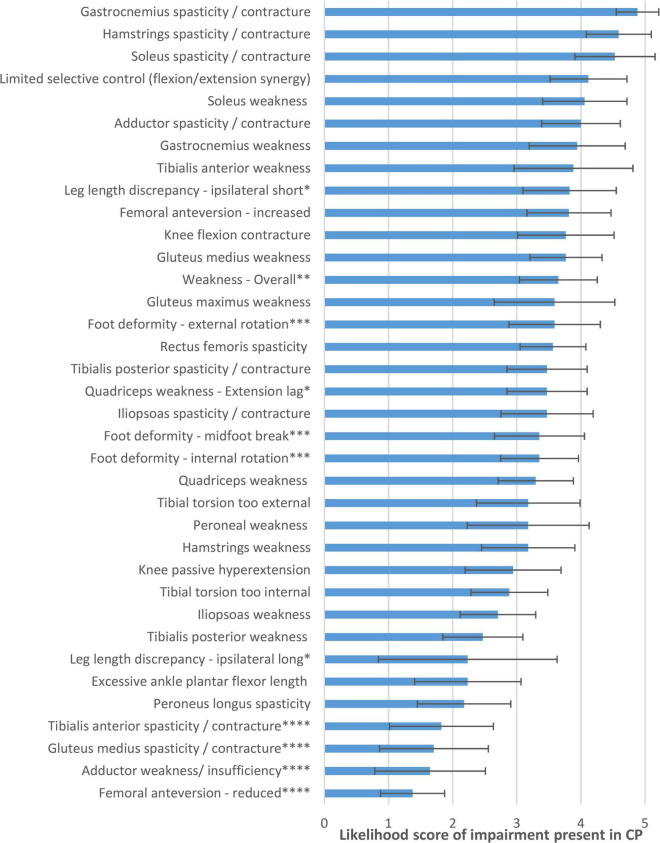
Likelihood of impairments being present in CP, as judged by the expert panel in questionnaire 2. ^*^Reworded in final list to “Leg length discrepancy, shortest/longest leg.” ^**^Excluded in final list, as too general to be coupled with specific gait features. ^***^Reworded/combined in final list, to “Foot deformity” only. ^****^Excluded in final list, as very unlikely in CP.

Figure 1 shows the flow chart describing how items were discussed, reworded, excluded, or added in the expert meetings. Figure 2 lists the likelihood of all impairments presented in Q2. For the 123 impairment-gait feature relations in Q3, the average likelihood was 3.6 ± 0.8 (median 3.6). The final set as based on Q4 and Q5 consisted of 120 gait feature–impairment relations. These items received an average likelihood score of 3.5 ± 0.6 points (median 3.6, range 2.1–4.6). This final set of all relevant gait feature–impairment relations is presented in Supplementary Appendix A, while Supplementary Appendix B contains the reversed set with impairment gait feature relations.

## Discussion

This study presents the development of a core set of gait features and potential underlying impairments to assist gait analysts and clinicians in standardized clinical reasoning in gait analysis for children with CP. A total of 120 gait feature–impairment relations received consensus on being likely in CP and were included. A unique aspect is that the tables are presented in both directions, e.g., to assist in searching for possible causes for a gait deviation (Supplementary Appendix A), or to help find potential gait features related to a specific impairment (Supplementary Appendix B).

**FIGURE 3:**
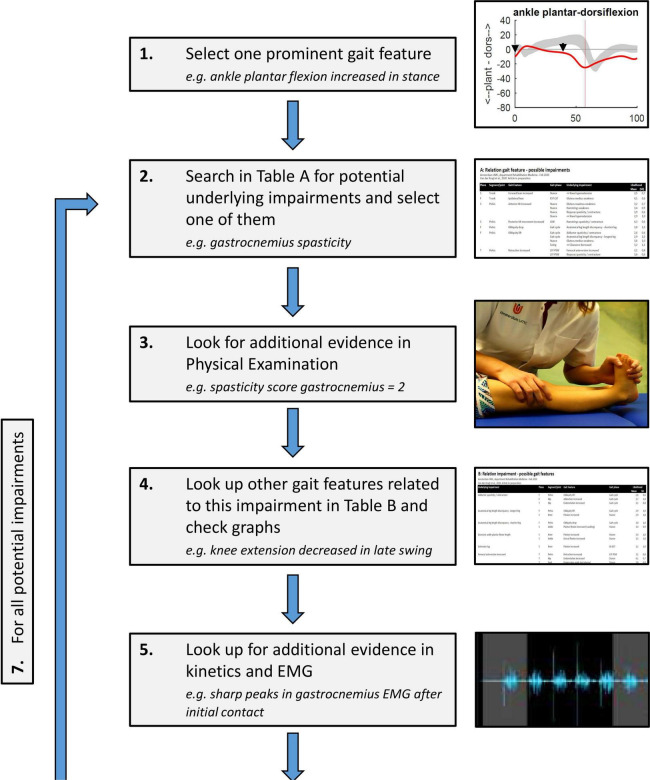
Stepwise process of clinical reasoning using the developed look-up tables in Supplementary Appendixes A, B: (1) One kinematic gait feature is chosen, preferably related to the clinical problem of the patient; (2) A potential underlying impairment is identified using the table in Supplementary Appendix A; (3) Physical exam data is checked to see whether this impairment was observed in clinical testing; (4) All other related gait features for this impairment are looked up using the table in Supplementary Appendix B; the more of these features are present in the patient’s gait, the likelier it is that the impairment has a large and relevant effect on gait; (5) Additional evidence for the role of the impairment during gait is searched in EMG, kinetics, etc. (6) If, based on steps 3–5, the impairment is considered likely, it can be added to the list of impairments affecting gait for the gait report; (7) The process is repeated in an iterative process from step (2) until all possible likely impairments for the selected gait feature are identified. After this, the process is repeated from step (1) for other relevant gait features, until all are solved.

The developed look-up tables can be used to assist the clinical reasoning process, following the impairment focused interpretation approach (Baker, 2013). After systematically listing all relevant kinematic gait features, the process of relating features to each other and linking these to underlying impairments could take the following stepwise approach, as exemplified in Figure 3:

1) From the list of kinematic gait features, one feature is chosen, preferably related to the clinical problem of the patient;
2) Potential underlying impairments for this feature are identified using the table in Supplementary Appendix A. One of these is chosen for further consideration (the choice of which is trivial, as all potential impairments will be checked throughout the process);
3) Physical exam data is checked to see whether this impairment was observed in clinical testing;
4) All other gait features that are related to this impairment are looked up using the table in Supplementary Appendix B; the more of these features are present in the patient’s gait, the likelier it is that the impairment has a large and relevant effect on gait;
5) Additional evidence for the role of the impairment during gait is searched in EMG data, kinetics, etc.;
6) If, based on steps 3–5 (i.e., the combination of gait features, physical exam data, EMG, kinetics and other supporting data), the impairment is considered likely, it can be added to the list of impairments affecting gait for the gait report.
7) This process is repeated in an iterative process from step (2) until all possible likely impairments for the selected gait feature are identified.

After this, the process can be repeated from step (1) for other relevant gait features, until all are solved. This process results in an overview of the most likely underlying impairments related to the patient’s walking problem, and how these impairments explain the combined features of the abnormal gait pattern.

Clearly, this suggested process is restricted to the neuromechanical approach of gait analysis interpretation. It aims to disentangle potential underlying neuromechanical impairments using a combination of biomechanical gait data with clinical findings, to arrive at potential targets for intervention to enhance walking ability. The initial evaluation on 20 patient cases indicated that the main features and impairments as present in CP were included, helping to perform the impairment-focused interpretation approach in a more systematic manner. Nevertheless, medical decision making can never be fully based on such an interpretation tool only. The decision on which identified underlying impairments are most relevant for the patient’s functional walking problems, and which should be treated, is still up to the expert clinician. Moreover other factors beyond the neuromechanical domain should be considered, including environmental and personal factors, as well as factors such as musculoskeletal pain, cognitive impairments, and visual or sensory deficits, that may also play an important role in walking problems.

This proposed process to identify all relevant impairments is intended to improve standardization and transparency of gait analysis interpretation. This opens up possibilities for more automated, computerized identification of features, and the development of software tools that can help to automatically relate these features to all potential underlying impairments. It is an explicit way of reasoning, where all decisions are consciously made by the clinician based on suggestions from the presented core set. This is in contrast to several more “black-box” machine learning approaches as recently proposed (e.g., Lai et al., 2009, Chia et al., 2020; Kidzinski et al., 2020). In future research, it would be interesting to compare the flow of reasoning and outcome of these different approaches, and potentially combine both approaches taking the user decisions into account when training a decision support system using artificial intelligence systems.

Although the look-up tables in this study were developed through a careful systematic process, several specific assumptions and simplifications had to be made throughout. First, it was chosen to combine the impairments spasticity and contractures into one item, as their effect on gait is difficult to disentangle based on kinematics alone. Hence, describing their related gait features separately would merely double the length of the tables with many repetitions. The clinically important distinction which of the two is most prevalent, should be made based on physical examination and/or EMG data. Nevertheless, subtle differences in effects on kinematics may exist between spasticity or contracture of the same muscle, which could be further specified in future studies. Second, although clinically important, more detailed (within-)foot and ankle deviations were not included in the present list to limit the scope, as they can be quite diverse and complex. An extension of the same approach, focusing specifically on foot and ankle deviations, would be a relevant future addition to this core set. Third, gait features were described in a qualitative manner, sometimes with quite broad phases of gait (e.g., all of stance or swing), in order to include all potential deviations over this period. Whether or not a gait feature should be considered abnormal and incorporated in the clinical reasoning process, is still up to the subjective expert view of the assessor. Finally, specific to the Delphi approach taken, the validity of the individual feature–impairment relationship was supported in this study by expert group consensus. For practical reasons and to ensure a somewhat similar tradition of gait data interpretation, it was chosen to perform the Delphi process with a national expert group. Next steps therefore include extension of the expert panel to international experts and include their views on data interpretation, as well as assessing the reliability of the proposed approach, and further validation of individual relationships based on experimental or simulation-based studies.

## Conclusion

This study was one of the first that aimed specifically to standardize and support the interpretation process of gait data in children with CP. The developed look-up tables, linking a core set of gait features and impairments based on literature, biomechanical reasoning and expert consensus, supports an impairment focused interpretation approach in a systematic and transparent manner. Future study can build on the developed set of look-up tables, by further assessing their validity and reliability, and by automating the process of gait feature selection and of linking these features to potential underlying impairments.

## Data availability statement

The raw data supporting the conclusions of this article will be made available by the authors after request.

## Ethics statement

For this study we used video footage and 3D gait reports of clinical gait analysis of children with CP performed during regular medical care. This study design was reviewed and approved by Medical Ethics Committee of Amsterdam UMC, The Netherlands. Written informed consent for their data to be used in scientific studies was provided by the participants’ legal guardians and all children above 11 years of age.

## The GAIT.SCRIPT study group

Barbara van Beeten, Christian Greve, Francisca Meuzelaar-Kiezebrink, Henrike van Proosdij, Herwin Horemans, Hurnet Dekkers, Katinka Folmer, Kenneth Meijer, Lenneke van Kats, Lucianne Speth, Marc Nederhand, Marie-Anne Kuiper, Peter Jongerius, and Yvonne Janssen-Potten.

## Author contributions

MK, HH, KW, KH, SD, and AB contributed to the conception and design of the study. SD and KW processed and organized the data and performed the statistical analysis. MK and SD wrote the final draft of the manuscript. All authors contributed to manuscript revision, read, and approved the submitted version.
