# Supplementary material for: Development of a core set of gait features and their potential underlying impairments to assist gait data interpretation in children with cerebral palsy
Source: Front Hum Neurosci. 2022 Oct 20;16:907565. doi: 10.3389/fnhum.2022.907565 (PMC9630336; doi:10.3389/fnhum.2022.907565)
Supplement: Supplementary file 1 [file Data_Sheet_1.pdf]

Appendix A: Relation gait feature - possible impairments

Developed at Amsterdam UMC, Department of Rehabilitation Medicine, as part of the GAIT.SCRIPT project

Supplementary material to: Van der Krogt et al. (2022), Frontiers in Human Neuroscience

Plane	Segment/joint	Gait Feature	Gait phase	Underlying impairment	Likelihood	
					Mean	(SD)
S	Trunk	Forward lean increased	Stance	<< Knee extension increased	3,5	0,7
S	Trunk	Hyper lordose	Stance	<< Anterior tilt increased	4,4	0,5
			Stance	<< Knee extension increased	3,4	0,8
F	Trunk	Ipsilateral lean	EST-LST	Gluteus medius weakness	4,1	0,6
S	Pelvis	Anterior tilt increased	Stance	Gluteus maximus weakness	3,2	0,7
			Stance	Hamstrings weakness	3,4	0,9
			Stance	Iliopsoas spasticity / contracture	3,9	0,6
			Stance	<< Knee extension increased	2,9	1,0
			Swing	Iliopsoas spasticity / contracture	3,6	1,0
S	Pelvis	Posterior tilt movement increased	LSW	Hamstrings spasticity / contracture	4,3	0,6
F	Pelvis	Obliquity drop	Gait cycle	Anatomical leg length discrepancy - shortest leg	3,0	1,0
F	Pelvis	Obliquity lift	Gait cycle	Adductor spasticity / contracture	2,6	0,6
			Gait cycle	Anatomical leg length discrepancy - longest leg	2,9	1,0
			Stance	Gluteus medius weakness	3,6	1,0
			Swing	<< Clearance decreased	3,2	1,1
T	Pelvis	Protraction increased	Stance	<< Contralateral retraction increased	3,8	0,7
T	Pelvis	Retraction increased	LST-PSW	Femoral anteversion increased	3,1	0,8
			LST-PSW	Iliopsoas spasticity / contracture	3,6	0,6
			LST-PSW	<< Hip extension decreased	3,9	0,7
S	Hip	Extension decreased	LST-PSW	Rectus femoris spasticity	2,6	1,0
			LST-PSW	Iliopsoas spasticity / contracture	3,8	0,6
			LST-PSW	Gluteus maximus weakness	2,7	1,3
S	Hip	Peak extension delayed	Stance	Gastrocnemius weakness	2,7	1,2
			Stance	Soleus weakness	2,8	1,3
			Stance	Gluteus maximus weakness	2,6	0,9
S	Hip	Flexion delayed	ESW	Limited selective control (flexion/extension synergy)	2,9	1,0
S	Hip	Flexion increased	Stance	Gluteus maximus weakness	3,1	1,0
			Stance	<< Knee flexion increased in stance	4,4	0,5
S	Hip	Flexion increased (high steps)	Swing	Limited selective control (flexion/extension synergy)	2,8	1,1
			Swing	<< Contralateral stance leg (functionally) shorter	2,9	1,0
S	Hip	Flexion decreased	Swing	Iliopsoas weakness	2,9	0,9
			Swing	<< Push-off decreased	3,9	0,9
F	Hip	Adduction increased	Gait cycle	Adductor spasticity / contracture	3,7	1,0
			Stance	Gluteus medius weakness	3,8	0,8
F	Hip	Abduction (circumduction)	Swing	<< Clearance decreased	3,8	0,9
			Swing	<< Contralateral stance leg (functionally) shorter	2,9	1,0
T	Hip	Exorotation increased	Gait cycle	Tibial torsion too internal	2,1	0,9
T	Hip	Endorotation increased	Gait cycle	Adductor spasticity / contracture	3,1	0,8
			Stance	Femoral anteversion increased	4,1	0,7
			Stance	Gluteus maximus weakness	2,9	0,8
			Stance	Tibial torsion too external	3,1	1,0
			LSW	Hamstrings spasticity / contracture	4,2	0,8
T	Hip	Adduction increased	LSW	Hamstrings spasticity / contracture	4,2	0,8
S	Knee	Extension decreased	LSW	<< Push-off decreased	3,5	0,9
			LSW	Gastrocnemius spasticity / contracture	3,3	1,0
			LSW	Hamstrings spasticity / contracture	4,1	0,7
			LSW	Limited selective control (flexion/extension synergy)	3,6	0,8
S	Knee	Extension increased	Stance	Quadriceps weakness	3,2	1,3
			EST-LST	Soleus spasticity / contracture	4,2	0,9
S	Knee	Extension movement	LR	Soleus spasticity / contracture	4,1	0,9
S	Knee	Flexion decreased	Swing	<< Reduced walking speed	3,4	0,7
			PSW-ESW	Iliopsoas weakness	2,6	1,1
			ESW	<< Knee flexion increased in LST-PSW	2,7	1,0
			ESW	Rectus femoris spasticity	3,6	0,6
			ESW	<< Push-off decreased	4,4	0,8
S	Knee	Flexion delayed	ESW	Limited selective control (flexion/extension synergy)	3,4	0,9
S	Knee	Flexion increased	Stance	Knee flexion contracture	4,1	1,0
			Stance	Excessive ankle plantar flexor length	3,4	1,0
			Stance	Foot deformity	3,6	0,7

Appendix A (continued): Relation gait feature - possible impairments

Plane	Segment/joint	Gait Feature	Gait phase **	Underlying impairment	Likelihood Mean	(SD)
S	Knee	Flexion increased (continued)	Stance	Gastrocnemius spasticity / contracture	3,6	1,3
			Stance	Gluteus maximus weakness	2,6	0,9
			Stance	Iliopsoas spasticity / contracture	3,2	0,9
			Stance	Limited selective control (flexion/extension synergy)	3,2	1,0
			Stance	Anatomical leg length discrepancy - longest leg	2,9	1,0
			EST-LST	Hamstrings spasticity / contracture	2,9	1,0
			EST	Gastrocnemius weakness	3,8	1,1
			LST	Gastrocnemius weakness	4,2	0,9
			LR-EST	Extension lag	3,1	1,0
			EST	Soleus weakness	4,0	1,0
		Flexion increased (high steps)	Swing	<< Contralateral stance leg (functionally) shorter	2,6	0,9
S	Tibia	Backward inclination	EST-LST	Soleus spasticity / contracture	3,9	0,9
			EST-LST	Quadriceps weakness	2,2	0,6
S	Tibia	Forward inclination increased (v)	EST-LST	Soleus weakness	4,3	0,8
			EST-LST	Gastrocnemius weakness	3,9	0,7
S	Ankle	Dorsal flexion increased	LR-LST	Excessive ankle plantar flexor length	3,5	1,0
			Stance	Knee flexion contracture	3,3	1,0
			LR-LST	Soleus weakness	4,5	0,6
			LST	Gastrocnemius weakness	4,2	0,6
S	Ankle	Plantar flexion decreased	PSW	Gastrocnemius weakness	4,4	0,6
			PSW	Soleus weakness	4,0	0,9
S	Ankle	Plantar flexion increased	LSW	Gastrocnemius spasticity / contracture	4,1	0,9
			LR-LST	Gastrocnemius spasticity / contracture	4,4	0,7
			LSW	Limited selective control (flexion/extension synergy)	3,7	0,9
			MST	Limited selective control (flexion/extension synergy)	2,9	0,8
			LSW	Soleus spasticity / contracture	3,1	1,0
			LR-LST	Soleus spasticity / contracture	4,1	0,7
			Swing	Tibialis anterior weakness	3,9	1,0
		Plantar flexion increased (vaulting)	Stance	Anatomical leg length discrepancy - shortest leg	3,4	0,9
S	Ankle	Plantar flexion peak too early	EST-LST	Gastrocnemius spasticity / contracture	4,6	0,6
			EST-LST	Soleus spasticity / contracture	4,3	0,8
T	Ankle	Internal rotation increased	Gait cycle	Tibialis posterior spasticity / contracture	3,3	1,1
			Gait cycle	Gastrocnemius spasticity / contracture	3,2	0,4
			Gait cycle	Peroneus weakness	3,2	0,8
F	Ankle	Inversion (varus) increased	Gait cycle	Tibialis posterior spasticity / contracture	3,9	0,7
			Gait cycle	Gastrocnemius spasticity / contracture	3,2	0,4
			Gait cycle	Peroneus weakness	3,2	0,8
F	Ankle	Eversion (valgus) increased	Gait cycle	Peroneus spasticity / contracture	2,2	0,9
			Gait cycle	Tibialis posterior weakness	3,3	0,6
S	Foot	Early heelrise (v)	EST	Gastrocnemius spasticity / contracture	4,5	0,6
			EST	Soleus spasticity / contracture	4,2	0,8
S	Foot	Toe walking (v)	Stance	Gastrocnemius spasticity / contracture	4,5	0,6
			Stance	Soleus spasticity / contracture	4,2	0,8
S	Foot	Forefoot/ midfoot contact (v)	IC	Tibialis anterior weakness	4,1	0,9
			IC	Limited selective control (flexion/extension synergy)	3,5	0,8
			IC	Gastrocnemius spasticity / contracture	4,2	0,9
			IC	Soleus spasticity / contracture	3,9	1,0
			IC	Foot deformity	3,2	0,8
S	Foot	Clearance decreased (v)	ESW	<< Ankle plantar flexion increased in ESW	3,9	1,2
			ESW	<< Knee flexion decreased in ESW	4,3	0,6
F	Foot	Lateral foot contact (v)	IC	Foot deformity	4,0	1,0
			IC	Gastrocnemius spasticity / contracture	2,6	0,9
			IC	Peroneus weakness	3,3	0,7
			IC	Tibialis posterior spasticity / contracture	4,5	0,5
F	Foot	Narrow stride width	Stance	Hamstrings spasticity / contracture	3,4	0,7
T	Foot	Progression angle too external	Stance	Foot deformity	4,0	0,7
			Stance	Tibial torsion too external	4,0	0,7
T	Foot	Progression angle too internal	Stance	Foot deformity	3,7	0,6
			Stance	Tibial torsion too internal	3,6	0,9
			Stance	Peroneus weakness	2,7	0,9
			Stance	Femoral anteversion increased	3,8	0,8

Abbreviations of gait events and phases: IC, initial contact; LR, loading response (~0-10% gait cycle); EST, early stance (~10-30%); MST, midstance (~30%); LST, late stance (30-50%); PSW, preswing (~50-60%); ESW, early swing (~60-80%); LSW, late swing (~80-100%)

(v): item can best be determined from video rather than from 3D motion capture data

<<: related to (indirect cause)
