# Supplementary material for: Development of a core set of gait features and their potential underlying impairments to assist gait data interpretation in children with cerebral palsy
Source: Front Hum Neurosci. 2022 Oct 20;16:907565. doi: 10.3389/fnhum.2022.907565 (PMC9630336; doi:10.3389/fnhum.2022.907565)
Supplement: Supplementary file 2 [file Data_Sheet_2.pdf]

Appendix B: Relation impairment - possible gait features

Developed at Amsterdam UMC, Department of Rehabilitation Medicine, as part of the GAIT.SCRIPT project

Supplementary material to: Van der Krogt et al. (2022), Frontiers in Human Neuroscience

Underlying impairment	Plane	Segment/ joint	Gait Feature	Gait phase	Likelihood	
					Mean	SD
Adductor spasticity / contracture	F	Pelvis	Obliquity lift	Gait cycle	2,6	0,6
	F	Hip	Adduction increased	Gait cycle	3,7	1,0
	T	Hip	Endorotation increased	Gait cycle	3,1	0,8
Anatomical leg length discrepancy - longest leg	F	Pelvis	Obliquity lift	Gait cycle	2,9	1,0
	S	Knee	Flexion increased	Stance	2,9	1,0
Anatomical leg length discrepancy - shortest leg	F	Pelvis	Obliquity drop	Gait cycle	3,0	1,0
	S	Ankle	Plantar flexion increased (vaulting)	Stance	3,4	0,9
Excessive ankle plantar flexor length	S	Knee	Flexion increased	Stance	3,4	1,0
	S	Ankle	Dorsal flexion increased	Stance	3,5	1,0
Extension lag	S	Knee	Flexion increased	LR-EST	3,1	1,0
Femoral anteversion increased	T	Pelvis	Retraction increased	LST-PSW	3,1	0,8
	T	Hip	Endorotation increased	Stance	4,1	0,7
	T	Foot	Progression angle too internal	Stance	3,8	0,8
Foot deformity	S	Knee	Flexion increased	Stance	3,6	0,7
	S	Foot	Forefoot/ midfoot contact (v)	IC	3,2	0,8
	F	Foot	Lateral foot contact (v)	IC	4,0	1,0
	T	Foot	Progression angle too external	Gait cycle	4,0	0,7
	T	Foot	Progression angle too internal	Gait cycle	3,7	0,6
Gastrocnemius spasticity / contracture	S	Knee	Extension decreased	LSW	3,3	1,0
	S	Knee	Flexion increased	Stance	3,6	1,3
	S	Ankle	Plantar flexion increased	LSW	4,1	0,9
	S	Ankle	Plantar flexion increased	LR-LST	4,4	0,7
	S	Ankle	Plantar flexion peak too early	EST-LST	4,6	0,6
	T	Ankle	Internal rotation increased	Gait cycle	3,2	0,4
	F	Ankle	Inversion (varus) increased	Gait cycle	3,2	0,4
	S	Foot	Early heelrise (v)	EST	4,5	0,6
	S	Foot	Toe walking (v)	Stance	4,5	0,6
	S	Foot	Forefoot/ midfoot contact (v)	IC	4,2	0,9
	F	Foot	Lateral foot contact (v)	IC	2,6	0,9
Gastrocnemius weakness	S	Hip	Peak extension delayed	Stance	2,7	1,2
	S	Knee	Flexion increased	EST	3,8	1,1
	S	Knee	Flexion increased	LST	4,2	0,9
	S	Tibia	Forward inclination increased (v)	EST-LST	3,9	0,7
	S	Ankle	Dorsal flexion increased	LST	4,2	0,6
	S	Ankle	Plantar flexion decreased	PSW	4,4	0,6
Gluteus maximus weakness	S	Pelvis	Anterior tilt increased	Stance	3,2	0,7
	S	Hip	Extension decreased	LST-PSW	2,7	1,3
	S	Hip	Peak extension delayed	Stance	2,6	0,9
	S	Hip	Flexion increased	Stance	3,1	1,0
	T	Hip	Endorotation increased	Stance	2,9	0,8
	S	Knee	Flexion increased	Stance	2,6	0,9
Gluteus medius weakness	F	Trunk	Ipsilateral lean	EST-LST	4,1	0,6
	F	Pelvis	Obliquity lift	Stance	3,6	1,0
	F	Hip	Adduction increased	Stance	3,8	0,8
Hamstrings spasticity / contracture	S	Pelvis	Posterior tilt movement increased	LSW	4,3	0,6
	T	Hip	Endorotation increased	LSW	4,2	0,8
	T	Hip	Adduction increased	LSW	4,2	0,8
	S	Knee	Extension decreased	LSW	4,1	0,7
	S	Knee	Flexion increased	EST-LST	2,9	1,0
	F	Foot	Narrow stride width	Stance	3,4	0,7
Hamstrings weakness	S	Pelvis	Anterior tilt increased	Stance	3,4	0,9

Appendix B (continued): Relation impairment - possible gait features

Underlying impairment	Plane	Segment/ joint	Gait Feature	Gait phase	Likelihood	
					Mean	SD
Iliopsoas spasticity / contracture	S	Pelvis	Anterior tilt increased	Stance	3,9	0,6
	S	Pelvis	Anterior tilt increased	Swing	3,6	1,0
	T	Pelvis	Retraction increased	LST-PSW	3,6	0,6
	S	Hip	Extension decreased	LST-PSW	3,8	0,6
	S	Knee	Flexion increased	Stance	3,2	0,9
Iliopsoas weakness	S	Hip	Flexion decreased	Swing	2,9	0,9
	S	Knee	Flexion decreased	PSW-ESW	2,6	1,1
Knee flexion contracture	S	Knee	Flexion increased	Gait cycle	4,1	1,0
	S	Ankle	Dorsal flexion increased	Stance	3,3	1,0
Limited selective control (flexion/extension synergy)	S	Hip	Flexion delayed	ESW	2,9	1,0
	S	Hip	Flexion increased (high steps)	Swing	2,8	1,1
	S	Knee	Extension decreased	LSW	3,6	0,8
	S	Knee	Flexion delayed	ESW	3,4	0,9
	S	Knee	Flexion increased	Stance	3,2	1,0
	S	Ankle	Plantar flexion increased	LSW	3,7	0,9
	S	Ankle	Plantar flexion increased	EST-LST	2,9	0,8
	S	Foot	Forefoot/ midfoot contact (v)	IC	3,5	0,8
Peroneus spasticity / contracture	F	Ankle	Eversion (valgus) increased	Gait cycle	2,2	0,9
Peroneus weakness	T	Ankle	Internal rotation increased	Gait cycle	3,2	0,8
	F	Ankle	Inversion (varus) increased	Gait cycle	3,2	0,8
	T	Foot	Progression angle too internal	Stance	2,7	0,9
	F	Foot	Lateral foot contact (v)	IC	3,3	0,7
Quadriceps weakness	S	Knee	Extension increased	Stance	3,2	1,3
	S	Tibia	Backward inclination	EST-LST	2,2	0,6
Rectus femoris spasticity	S	Hip	Extension decreased	LST	2,6	1,0
	S	Knee	Flexion decreased	ESW	3,6	0,6
Soleus spasticity / contracture	S	Knee	Extension movement	LR	4,1	0,9
	S	Knee	Extension increased	EST-LST	4,2	0,9
	S	Tibia	Backward inclination	EST-LST	3,9	0,9
	S	Ankle	Plantar flexion increased	LSW	3,1	1,0
	S	Ankle	Plantar flexion increased	LR-LST	4,1	0,7
	S	Ankle	Plantar flexion peak too early	EST-LST	4,3	0,8
	S	Foot	Early heelrise	EST	4,2	0,8
	S	Foot	Toe walking (v)	Stance	4,2	0,8
	S	Foot	Forefoot/ midfoot contact (v)	IC	3,9	1,0
Soleus weakness	S	Hip	Peak extension delayed	Stance	2,8	1,3
	S	Knee	Flexion increased	EST	4,0	1,0
	S	Tibia	Forward inclination increased (v)	EST-LST	4,3	0,8
	S	Ankle	Dorsal flexion increased	LR-LST	4,5	0,6
	S	Ankle	Plantar flexion decreased	PSW	4,0	0,9
Tibial torsion too external	T	Hip	Endorotation increased	Stance	3,1	1,0
	T	Foot	Progression angle too external	Stance	4,0	0,7
Tibial torsion too internal	T	Hip	Exorotation increased	Gait cycle	2,1	0,9
	T	Foot	Progression angle too internal	Stance	3,6	0,9
Tibialis anterior weakness	S	Ankle	Plantar flexion increased	Swing	3,9	1,0
	S	Foot	Forefoot/ midfoot contact (v)	IC	4,1	0,9
Tibialis posterior spasticity / contracture	T	Ankle	Internal rotation increased	Gait cycle	3,3	1,1
	F	Ankle	Inversion (varus) increased	Gait cycle	3,9	0,7
	F	Foot	Lateral foot contact (v)	IC	4,5	0,5
Tibialis posterior weakness	F	Ankle	Eversion (valgus) increased	Gait cycle	3,3	0,6

Abbreviations of gait events and phases: IC, initial contact; LR, loading response (~0-10% gait cycle); EST, early stance (~10-30%); MST, midstance (~30%); LST, late stance (30-50%); PSW, preswing (~50-60%); ESW, early swing (~60-80%); LSW, late swing (~80-100%)

(v): item can best be determined from video rather than from 3D motion capture data
